# A Roadmap From Feasibility to Reality: Can Greece Establish and Sustain a Transplant-Hepatopancreatobiliary (HPB) Fellowship?

**DOI:** 10.7759/cureus.94291

**Published:** 2025-10-10

**Authors:** Dimitrios Moris, Nikolaos Dimitrokallis, Emmanouil Giorgakis

**Affiliations:** 1 Department of Surgery, Duke University Medical Center, Durham, USA; 2 1st Department of Surgery and Transplantation, Evangelismos General Hospital, Athens, GRC; 3 Surgery, University of North Carolina at Chapel Hill, Chapel Hill, USA

**Keywords:** fellowship training, future educational reform, greece, surgical training programme, transplant and hepatobiliary surgeon

## Abstract

Hepatopancreatobiliary (HPB) and transplant surgery in Greece has expanded significantly, with high-volume centers in Athens and Thessaloniki achieving outcomes that meet international benchmarks. Despite adequate case volume, no structured fellowship pathway exists, driving many surgeons abroad and perpetuating workforce attrition. We assessed the feasibility of establishing a national transplant-HPB fellowship in Greece and defined key structural requirements. We reviewed national case volumes in HPB and transplant surgery, benchmarked outcomes against international standards, and identified systemic barriers to fellowship development. We then developed a proposed framework for a national consortium fellowship, including curriculum design, accreditation pathways, funding strategies, and faculty recruitment. Barriers include a lack of formal accreditation mechanisms, fragmented case distribution, insufficient protected faculty time, and limited integration of robotics and simulation.

A roadmap for implementation includes (1) creation of a national training consortium; (2) adoption of competency-based curricula aligned with International Hepato-Pancreato-Biliary Association (IHPBA)/American Society of Transplant Surgeons (ASTS) standards; (3) diversified funding through the National Health System, universities, foundations, and professional societies; and (4) repatriation of internationally trained faculty to strengthen mentorship and accreditation compliance. Greece possesses sufficient case volume and expertise to support an accredited transplant-HPB fellowship. Establishing a national program through resource consolidation, diversified funding, and international collaboration would not only mitigate surgical brain drain but also position Greece as a regional training hub in advanced HPB and transplant surgery.

## Editorial

Introduction

Over the past decade, surgical practice in Greece has undergone a silent transformation. High-volume hepatopancreaticobiliary (HPB) and transplant services now operate in Athens and Thessaloniki, performing complex resections and multi-organ transplants with outcomes that meet international benchmarks [1]. In 2024, the Hellenic Transplant Organization reported over 50 liver transplants nationwide, with parallel growth being observed in liver and pancreatic surgery, where annual volumes of pancreatectomies in certain high-volume centers exceed 80 cases, a threshold recognized by the International Hepato-Pancreato-Biliary Association (IHPBA) as conducive to advanced fellowship training [1,2].

Despite this progress, Greece lacks a structured, accredited fellowship pathway in transplantation and HPB. As a result, talented surgeons have to train abroad, often not returning, exacerbating the surgical “brain drain” [3,4]. Meanwhile, the national health system continues to rely on a small cadre of overextended specialists.

The current landscape: clinical volume and complexity

The sustainability of a fellowship hinges on procedural volume and complexity. Greek high-volume tertiary centers and university hospitals collectively perform an estimated 120-150 liver resections, 50 liver transplants, 100-120 pancreatoduodenectomies, and 70 distal or total pancreatectomies annually [5-7]. These figures, while modest compared to high-income countries with large catchment areas, are competitive within the European Union when aggregated in a coordinated training network [8,9].

Pancreatic surgery, in particular, has seen a steady rise in both volume and complexity, driven by improved perioperative care and multidisciplinary oncology pathways. Outcomes for elective pancreatic resections in experienced Greek units are comparable to European benchmarks, with 90-day mortality rates of 3-5% and median lymph node yields meeting oncological standards [5,10]. These data underscore that the technical case mix exists to support the hands-on component of fellowship training, especially if centers agree to pool cases under a unified fellowship curriculum.

Overview of barriers to the establishment of a Transplant-HPB Fellowship

Several challenges stand in the way of establishing a Greek Transplant-HPB fellowship. First, the absence of a fellowship program is not due to a lack of cases or expertise, but rather systemic barriers. There is no central accreditation body within Greece aligned with global surgical education standards, making local recognition difficult. Second, faculty development and program directorship positions lack protected time and dedicated funding. Third, international visibility of Greek surgical units remains low due to limited engagement in multicenter research and inadequate public outreach. Also, without a transparent fellow-matching process, concerns about equity and meritocracy deter applicants. Moreover, funding constraints in the public health sector may limit investment in robotics, simulation, and dedicated fellowship salaries. Furthermore, current surgical training structures in Greece lack formal post-residency subspecialty accreditation, creating regulatory ambiguity. The geographic dispersion of high-volume cases among multiple hospitals can fragment exposure unless a deliberate case-sharing network is created. Finally, the entrenched apprenticeship model, while strong in its traditions, requires cultural adaptation toward structured curricula, objective assessment, and competency milestones.

Technology integration: the case for robotics and advanced platforms

While Greek HPB and transplant units have developed expertise in conventional open approaches, and some have in laparoscopic approaches, robotics adoption remains uneven, with only a few centers equipped with the Da Vinci Xi (Intuitive Surgical, Inc., Sunnyvale, California, US) or equivalent systems. Internationally, robotics is becoming central to HPB and transplant training, particularly in complex hilar dissections, living donor hepatectomy, and minimally invasive pancreatoduodenectomy [11]. Incorporating robotics into the fellowship would not only align with global trends but also serve as a recruitment incentive for highly skilled Greek surgeons abroad. Investment in simulation platforms, wet lab training, and proctor-led robotic modules would be essential to meet competency-based learning objectives. However, cases remain concentrated in a few units, with no coordinated national rotation system [12]. 

Step-by-step roadmap to program creation

The creation of a national Transplant-HPB fellowship should proceed in defined stages. The path forward begins with forming a national HPB-transplant training consortium, comprising the major academic and transplant units. This consortium should submit a joint application for European fellowship accreditation, pooling combined case logs to surpass minimum thresholds of 50 major liver resections, 20-30 liver transplants, and 40-50 major pancreatic resections per fellow per year [13,14]. Initially, a consortium of the country’s leading centers must be formed under the auspices of the Hellenic Surgical Society and the Hellenic Transplant Organization. This body would define a unified curriculum modeled after IHPBA/American Society of Transplant Surgeons (ASTS) competencies. Simultaneously, the program must secure Ministry of Health recognition and seek IHPBA endorsement. Recruitment should prioritize repatriating experienced Greek faculty from abroad, particularly those holding international fellowship training and active academic portfolios.

Didactics should be structured weekly, covering surgical anatomy, operative planning, transplant immunology, oncology principles, and advanced technology training (including robotics and laparoscopic simulation). Research should be embedded through mandatory participation in ongoing clinical or translational projects, with fellows presenting at least one paper at an international congress annually. Finally, the fellow selection process should follow a transparent, match-style format with objective scoring based on academic merit, interview performance, and letters of recommendation.

Faculty excellence: leveraging the Greek diaspora

The most successful Transplant-HPB fellowships globally are defined not only by case volume but also by mentorship quality. Greece has a substantial diaspora of Greek-trained surgeons holding faculty positions in leading North American and European HPB and transplant centers. Strategic repatriation of such faculty through competitive contracts, protected academic time, and research infrastructure could rapidly elevate the training environment. Internationally trained Greek faculty could also bridge accreditation requirements from the European Union of Medical Specialists (UEMS), Division of HPB Surgery, and the European Board of Surgery, ensuring the program meets cross-border training standards [14].

Funding

The funding strategy for a Greek Transplant-HPB fellowship relies on a diversified model that balances core support from the public healthcare system with supplemental contributions from academic institutions, private foundations, and international societies. The fellow’s salary and basic clinical training costs would primarily be covered by the ESY (National Healthcare System) and the Ministry of Health, reflecting local salary scales, while university hospitals would provide infrastructure, laboratory resources, and partial research support. Private foundations, such as the Onassis and Stavros Niarchos Foundations, could contribute to research projects, housing, and travel, ensuring the fellow has access to necessary resources beyond clinical duties [15,16]. International societies and professional organizations, including IHPBA and the European-African Hepato-Pancreato-Biliary Association (EAHPBA), would provide targeted grants for observerships, workshops, and conference participation. Small-scale industry support could cover consumables or procedural training costs, completing a flexible, resilient funding framework that enables high-quality fellowship training within the financial realities of Greece.

Research, extroversion, and collaborations

Beyond operative training, the fellowship must foster academic productivity. Greek Transplant and HPB units already contribute to multicenter studies internationally [5,6,17], but participation remains sporadic. A formal research office within the fellowship program could coordinate data submission to registries, initiate investigator-led trials, and facilitate joint projects with institutes such as the Biomedical Research Foundation of the Academy of Athens, which could offer translational research opportunities in immunology, oncology, and regenerative medicine [18]. Also, a fellowship could formalize research rotations with dedicated protected time, grant-writing workshops, and mandatory conference presentations (EAHPBA, International Liver Transplantation Society (ILTS), European Society of Organ Transplantation (ESOT), and American Transplant Congress). Publication benchmarks, such as ≥2 peer-reviewed manuscripts per year, could be a requirement for graduation.

Extroversion-actively reaching beyond national borders-is essential. Hosting annual Transplant and HPB symposia in Greece, inviting visiting professors, and offering short-term observerships to international trainees can position the program as a regional hub.

International collaboration could be formalized via Memoranda of Understanding (MOUs) with high-volume Transplant-HPB centers in Europe and North America [19], enabling short-term rotations, shared educational webinars, and reciprocal faculty visits. Such partnerships would help benchmark outcomes, align training standards, and provide fellows with a broader exposure to techniques and systems-based practice (Table 1).

**Table 1:** Core infrastructure and requirements for a Greek Hepatopancreaticobiliary–Transplant fellowship MOUs: Memoranda of Understanding

Domain	Current Status in Greece	Target for Fellowship Launch	Feasibility in the Greek Network
Annual Major Hepatic Resections	>120 in leading centers	Maintain ≥100 per year	Achievable
Annual Pancreatectomies	80–90 in top units	Maintain ≥70 per year	Achievable
Annual Liver Transplants	50 nationwide	≥20-30 per fellow	Achievable
Robotic HPB Surgery	Minimal	≥30% elective resections robotic exposure	Needs Improvement
Faculty	Limited internationally trained faculty	Recruit ≥3 repatriated Greek faculty with fellowship experience	Needs Improvement
Didactics	Informal, center-specific	Weekly structured national curriculum	Needs Improvement
Research Output	Sporadic multicenter participation	≥2 peer-reviewed publications per fellow/year	Achievable
Fellow Selection	Ad hoc	Transparent national match process	Needs Improvement
International Collaborations	Occasional individual MOUs	Formalized institutional agreements	Needs Improvement

Conclusions

The creation of a Greek Transplant-HPB fellowship is not only feasible but timely. Greece possesses the surgical case volume, clinical expertise, and academic heritage to support an international Transplant-HPB fellowship. The current barriers are not technical, but structural and organizational. By uniting leading centers, recruiting faculty from the diaspora, standardizing training requirements, embedding robust didactics and research, and engaging internationally, Greece can transform from a training consumer to a regional training hub in advanced Transplant-HPB surgery. The establishment of such a program would not only retain talent but also elevate the country’s position in the global surgical community.
